# Diversity and phylogenetic relationships of *Glossina* populations in Nigeria and the Cameroonian border region

**DOI:** 10.1186/s12866-018-1293-6

**Published:** 2018-11-23

**Authors:** Stephen Saikiu Shaida, Judith Sophie Weber, Thaddeus Terlumun Gbem, Sen Claudine Henriette Ngomtcho, Usman Baba Musa, Mbunkha Daniel Achukwi, Mohammed Mamman, Iliya Shehu Ndams, Jonathan Andrew Nok, Soerge Kelm

**Affiliations:** 1 Nigerian Institute for Trypanosomiasis Research, Kaduna, Nigeria; 2 Centre for Biomolecular Interactions, University of Bremen, 28334 Bremen, Germany; 3 Department of Biology, Ahmadu Bello University, Zaria, Nigeria; 4 Africa Centre of Excellence for Neglected Tropical Diseases and Forensic Biotechnology, Ahmadu Bello University, Zaria, Nigeria; 5 Department of Biological Sciences, University of Ngaoundéré, P.O. Box 454, Ngaoundéré, Cameroon; 6 TOZARD Research laboratory, P.O.Box 59 Bambili-Tubah, Bamenda, Cameroon; 7 Department of Zoology, Ahmadu Bello University Zaria, Zaria, Nigeria; 8 Department of Biochemistry, Ahmadu Bello University Zaria, Zaria, Nigeria

**Keywords:** *Glossina* sp., *Glossina palpalis palpalis*, *Glossina morsitans submorsitans*, *Glossina tachinoides*, COI, *Glossina* populations, ITS-1

## Abstract

**Background:**

Tsetse flies are vectors of trypanosomes, parasites that cause devastating disease in humans and livestock. In the course of vector control programmes it is necessary to know about the *Glossina* species present in the study area, the population dynamics and the genetic exchange between tsetse fly populations.

**Results:**

To achieve an overview of the tsetse fly diversity in Nigeria and at the Nigeria-Cameroon border, tsetse flies were trapped and collected between February and March 2014 and December 2016. Species diversity was determined morphologically and by analysis of Cytochrome C Oxidase SU1 (COI) gene sequences. Internal transcribed spacer-1 (ITS-1) sequences were compared to analyse variations within populations. The most dominant species were *G. m. submorsitans*, *G. tachinoides* and *G. p. palpalis*. In Yankari Game Reserve and Kainji Lake National Park, *G. submorsitans* and *G. tachinoides* were most frequent, whereas in Old Oyo National Park and Ijah Gwari *G. p. palpalis* was the dominant species. Interestingly, four unidentified species were recorded during the survey, for which no information on COI or ITS-1 sequences exists. *G. p. palpalis* populations showed a segregation in two clusters along the Cameroon-Nigerian border.

**Conclusions:**

The improved understanding of the tsetse populations in Nigeria will support decisions on the scale in which vector control is likely to be more effective. In order to understand in more detail how isolated these populations are, it is recommended that further studies on gene flow be carried out using other markers, including microsatellites.

## Background

Tsetse flies (*Glossina* sp.) are the main vectors of trypanosomes, protozoan parasites that cause human African trypanosomosis (HAT) and animal African trypanosomosis (AAT) in livestock in sub-Saharan Africa. HAT cases are on the decline during the last decade to below 10,000 cases per year [1], however, sleeping sickness remains endemic in several countries, including Nigeria [2]. AAT is resulting in annual losses of approximately 5 billion US Dollars due to restricted agricultural development and livestock production [3]. The disease thus poses a big socioeconomic burden on sub-Saharan African countries.

Trypanosomes rely on tsetse flies as vectors during their infectious life cycle, where they develop into mammalian infective forms. Control and elimination of the vector is therefore considered the most appropriate technique for disease management [4].

The genus *Glossina* comprises 33 species and subspecies of tsetse flies (Diptera: Glossinidae) distributed across 38 countries of sub-Saharan Africa. The genus is split into three subgenera, *Glossina* (*Morsitans* group), *Nemorhina* (*Palpalis* group) and the *Austenina* (*Fusca* group) based on differences in their morphological characteristics and habitat preferences [5–7]. This has been confirmed by molecular analysis [8]. Members of the *Glossina* (*Morsitans*) and *Nemorhina* (*Palpalis*) groups are main vectors of AAT in livestock and HAT in humans respectively, and thus cause great public health hazard [9–11]. In contrast, members of the *Austenina* (*Fusca* group) are predominantly inhabitants of the tropical forests and considered to be of less economic importance due to their restricted distribution [9].

Presently, control campaigns against the disease vectors are limited to sequential aerial application of insecticides, pour-on formulations, impregnated screens and use of sterile insect technique (SIT) for *mop up* operations [12–14]. A major problem faced by all these strategies is reinvasion of cleared areas by other tsetse populations. Therefore, identification of the *Glossina* vector species and understanding of their population dynamics are core to control success and effective control measures against the vectors [15, 16]. This is especially important in the application of SIT, where sterile males are released in an existing population. Where extensive migration occurs, SIT may not succeed, but where there is limited migration, SIT may succeed [17].

Dispersal of tsetse fly populations can be monitored indirectly by studies on gene flow [18]. Genetic analysis of tsetse populations is important in determining isolation status and the likelihood of reinvasion of controlled areas by surrounding tsetse fly populations [19, 20]. Several studies have indicated that tsetse populations are genetically structured [21, 22]. Especially *G. p. palpalis,* clustering has been observed with distinct West African and Central African clades [8, 15, 23, 24], however, very few of such data are available on tsetse populations in Nigeria [15, 17, 18, 25, 26], where these two clades could meet as the country links West and Central Africa.

Game parks hold the highest populations of tsetse flies from where they disperse at periods of high population densities, as has been observed by studies in Nigeria [27, 28]. Some sleeping sickness cases have been linked to their closeness to national parks [29, 30]. Therefore, control of tsetse flies in National Parks will no doubt improve human and animal health and will boost agricultural production and livestock development in the country.

The aim of the study was to investigate the diversity of *Glossina* species in Nigeria in National Parks and Game Reserves as well as at the Cameroon - Nigerian border of the Adamawa region, as reservoirs for tsetse populations during dry season. Cytochrome C Oxidase SU1 (COI) and Internal Transcribed Spacer-1 (ITS-1) sequences were used as molecular markers for overall population structure, as well as morphometric analysis of wing landmarks. Information obtained in this study provides the first countrywide picture of tsetse populations in Nigeria in at least five decades that will aid the choice of effective anti-tsetse intervention strategies by Nigerian authorities and the Pan African Tsetse and Trypanosomiasis Eradication Campaign (PATTEC) in Nigeria.

## Methods

### Description of study areas

Tsetse flies were collected from various highly tsetse infested areas in Nigeria (Table 1), among these three National Parks. The sampling sites were located several hundred kilometres from each other ranging from the forest, derived savannah to southern guinea savannah geo-ecological zones [31]. The survey was conducted during the end of the dry season March 2014. In Cameroon, samples were collected during March 2014 [32] and March and December 2016.

**Table 1:** GPS coordinates of the base camps of sampling areas in Nigeria (A) and Cameroon (B)

	GPS Coordinates	
	N	E
A. Nigeria sampling locations- Base Camps		
1 Yankari Game Reserve	9° 45.240’	10° 30.448’
2 Kainji Lake National Park	9° 53.832’	3° 59.140’
3 Old Oyo National Park	8° 25.051’	3° 46.726’
4 Cross River National Park Akamkpa	5° 21.829’	8° 26.180’
5 Cross River National Park Butateng	6° 16.433’	9° 07.901’
6 Ijah Gwari	9° 18.860’	7° 26.814’
B. Cameroon sampling location- Base Camp		
7 Dodeo Region	7° 27.994’	12° 04.101’

Yankari Game Reserve is situated in Bauchi State within the Northern Guinea/Sudan savannah vegetation zone. It covers an area of 2244 km^2^ and is located between latitude 9° 45′ N and longitude 10° 30′ E. Kainji Lake National Park spans an area of 5340 km^2^ across Niger and Kwara States (10° 22′ N and 4° 33′ E), within the southern guinea savannah vegetation zone. Old Oyo National Park is located in the northern part of Oyo State, South Western Nigeria. It has a total land area of 2512 km^2^. The Park lies within the derived savannah vegetation zone between latitudes 8° 10′ and 9° 05′ N and longitudes 3° 00′ and 4° 02′ E. Cross River National Park is located in the rainforest ecological zone in the extreme South East of Nigeria on the border with Republic of Cameroon. The park occupies a total land area of about 4000 km^2^ of tropical rain forest ecosystem, which thins out progressively into montane vegetation at the edge of the Obudu Plateau in Okwangwo area. Geographically it exists as two non-contiguous divisions, Oban and Okwangwo. The Southern Oban sector has an area of 3000 km^2^ while the Northern Okwango Division near Obudu covers an area of 1000 km^2^. The Park lies between latitude 5° 05′ and 6° 29′ N and longitude 8° 15′ and 9° 30^′^ E [33]. Ijah Gwari (near Suleja) is located between latitude 9° 12′ N and 9° 24′ N and longitude 7° 12′ E and 7° 20′ E in Tafa Local Government Area of Niger State. Several small streams traverse the area and the vegetation is riverine fringing forest forming a dense two-storey canopy.

The sampling site in Cameroon is Dodeo, close to the Nigerian border, located in Adamawa at latitude 7° 27′ N and longitude 12° 04′ E. Vegetation of the trapping sites was characterised by gallery forest along rivers.

### Tsetse sampling

Adult tsetse flies were collected using standard biconical traps [34] supplied by Vestagaard Frandsen. Ten to 18 traps were deployed at distances of at least 100 m intervals in riparian vegetation along the banks of rivers or streams and were continuously harvested for 48 h before redeployment. Flies were collected after every 24 h and all catches were harvested and transferred into cool boxes for conveyance to the base camp for sorting, physical examination and dissections. Each trap position was geo-referenced using a GPS device (GPSMAP® 60CSx Garmin). Ambient temperature and relative humidity were recorded at each trap position using a thermohygrometer during trap setting and harvesting of flies. Trap catches were sorted according to species, sex and nutritional status (teneral or non-teneral). *Glossina* species were identified morphologically using species identification keys [6, 35]. Fly apparent densities were calculated as number of flies per trap per day (F/T/D).

Only live flies were dissected and wings, legs, proboscis, salivary glands and gut were collected. Wings were stored dry for geometric morphometrics. Legs, proboscis and salivary glands were preserved in 200 μL nucleic acid preservation agent (NAPA: 25 mM sodium citrate, 10 mM EDTA, 70 g ammonium sulfate/100 mL solution, pH 7.5) in 1.5 mL cryotubes. Gut tissues were homogenized in 200 μL 50 mM Tris-HCl pH 9.0 by vortexing for 1 minute with four 2.38 mm metal beads (MoBio Laboratories, Carlsbad, California, USA). 50 μL of the homogenate was then added to 500 μL NAPA and preserved for subsequent DNA extraction. All collected tissues were kept cold and at − 20 °C when available. Long-term storage was at − 80 °C.

### DNA extraction

DNA was extracted from gut samples in NAPA using the Qiagen DNeasy Blood and Tissue Kit (Qiagen, Germany) following the manufacturer’s instructions and quantified using Nanodrop 1000 apparatus (Thermo Scientific-Germany) at a wavelength of 260 nm. DNA extraction from proboscis was done as described before [32] by grinding the proboscis with a mortar in 50 μL 10 mM Tris pH 8.0/ 0.5 mM EDTA. Extracted DNA was stored at − 20 °C until used.

### Molecular species identification

Partial gene fragments of COI and ITS-1 were amplified from purified gut DNA as described in [8]. PCR reactions were carried out in 25 μL final volume containing 2.5 μL 10× Dream*Taq* Green Buffer, 0.5 μL 10 mM dNTPs, 0.5 μL Dream*Taq* Polymerase (all supplied by Thermo Scientific, Dreieich, Germany), 0.5 μL 100 μM primers and 5.0 μL DNA template.

CO1 was amplified with CO1-forward (5′ TTG ATT TTT TGG TCA TCC AGA AGT-3′) and CO1-reverse (5′-TGA AGC TTA AAT TCA TTG CAC TAA TC-3′) primers, with an initial denaturation step at 95 °C for 5 min, followed by 35 cycles of 1 min at 94 °C, 1 min at 55 °C and 2 min at 72 °C. The final elongation was 10 min at 72 °C.

The ITS-1 region was amplified with ITS-1-forward (5′-GTG ATC CAC CGC TTA GAG TGA-3′) and ITS-1-reverse (5’-GCA AAA GTT GAC CGA ACT TGA-3′) primers, with an initial denaturation step at 95 °C for 5 min, followed by 30 cycles of 1 min at 94 °C, 1 min at 60 °C and 1.5 min at 72 °C. The final elongation was 7 min at 72 °C.

PCR amplicons were visualized in 1–2% agarose gel stained with G- Stain (Serva, Heidelberg, Germany) in TAE- Buffer (40 mM Tris, 10 mM Na-acetate, 1 mM EDTA, pH 8.0).

### DNA purification and sequencing

PCR amplicons were purified using GeneJet DNA purification kit (Thermo Scientific, Dreieich, Germany) following the manufacturer’s instruction and used for direct sequencing at the Max Planck Institute for Marine Microbiology-Bremen, Germany and by SeqLab, Göttingen, Germany.

### Data analysis

Obtained sequences were subjected to BLAST searches at the National Center for Biotechnology Information (NCBI) data base (Genbank). Sequences were analysed using Geneious Pro Version 5.5.9 and aligned by the Geneious Alignment algorithm, using the cost matrix for 93% similarity with a Gap open penalty of 12 and Gap extension penalty of 3. Phylogenetic analysis was performed using MEGA 6.0. Alignments were calculated with Muscle alignment tool (Gap open penalty − 400, Gap extension 0, Clustering methods (all iterations) UPGMB). Maximum likelihood trees were calculated using the model finder tool of MEGA. Tamura-Nei-93-model assuming gamma distribution with invariant sites using 900 bootstrap replicates was selected for COI sequence alignments of a 592 bp stretch of selected COI field sample representatives and reference. Tamura-3-parameter model with gamma distribution was selected with the default setting for number of discrete gamma categories and 800 bootstrap replicates for comparison of a 563 bp stretch of field samples. Complete gap deletion was employed for all COI samples.

ITS-1 sequences were analysed as described above, using pairwise gap deletion due to inherit variations in lengths.

### Geometric wing morphometrics

Forty-one individual *G. p. palpalis* flies collected at the border region in Cameroon had wings in a state suitable for morphometric analysis. Wings were secured on a microscope slide using a drop of 1:100 diluted Faure’sche solution (33.3 mL distilled water, 13.3% *v*/v glycerol, 20 g gum arabicum, 33.3% v/v chloralhydrate) on the wing root. They were covered with a cover glass and dried overnight. Pictures were taken using a Olympus SZX16 binocular (Olympus, Hamburg, Germany) with upward light (Olympus KL1500 LCD, Olympus, Hamburg, Germany) employing 10× magnification and scale bar added using Cell^F program (Olympus, Hamburg, Germany). Nine landmarks were selected for analysis using the CLIC package [36] as described previously [37]. COO was used to digitalize the landmarks. Procrustes superimposition (centring of configuration of landmarks, scaling, rotation) and comparison of centroid sizes was done using MOG. Samples were segregated according to their genetic group or sex.

## Results

### Tsetse fly collection

**Fig. 1:**
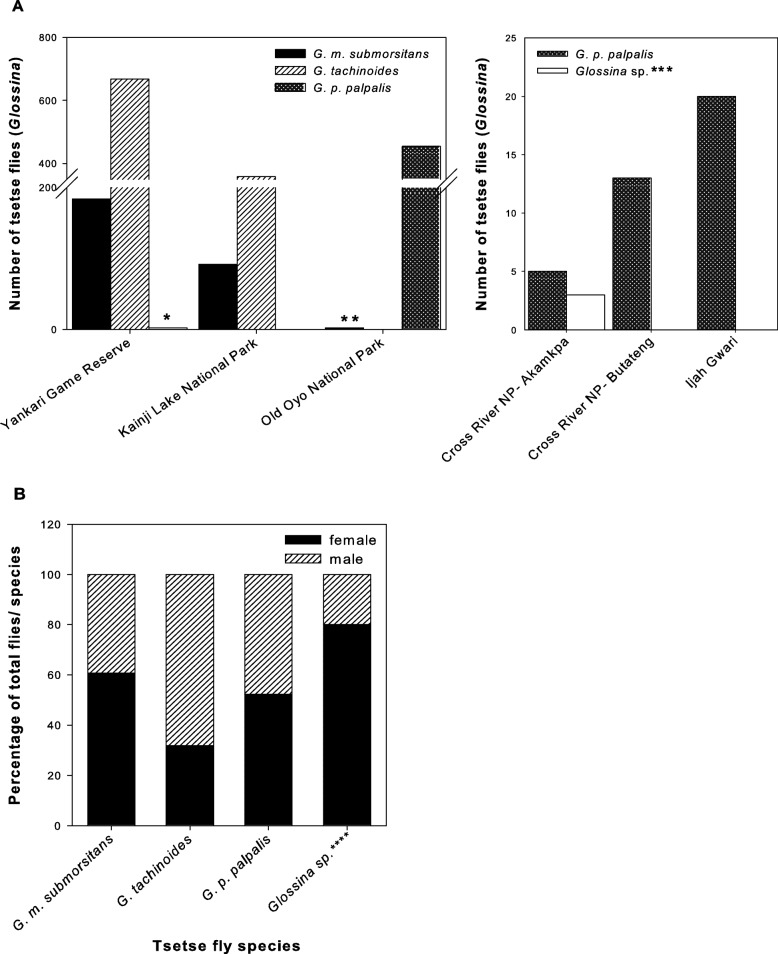
Total tsetse fly catches and sex ratio in Nigeria. All trapped tsetse flies regardless whether alive or dead are shown. A. Morphologically identified *Glossina* species are segregated according to the sampling region. Highest number of fly catches occurred in Yankari Game Reserve, Kainji Lake National Park and Old Oyo National Park (**a**). Low numbers of tsetse flies were caught in Cross River National Park and from Ijah Gwari (**b**). B. Sex ratio within the different species. *Two *G. p. palpalis* were collected in Yankari Game Reserve. **Two *G. m. submorsitans* from Old Oyo NP were later genetically identified as *Glossina* sp. ***Tsetse flies morphologically recorded as *G. fusca* were genetically identified as various *Glossina* species and were further referred to as *Glossina* sp. ****Glossina sp. refers to all unidentified *Glossina* species

To provide an overview of the *Glossina* species present in Nigeria during the end of dry season, six collection areas were visited from 22nd February to 19th March 2014. A total of 1802 tsetse flies were captured and morphologically identified (Fig. 1a). The most prevalent tsetse fly species were represented in the various sampling sites in Nigeria. *G. m. submorsitans* and *G. tachinoides* were found in Yankari Game Reserve and Kainji Lake National Park. *G. p. palpalis* was the predominant species in Old Oyo National Park and Ijah Gwari, while in Yankari Game Reserve only 2 specimens of *G. p. palpalis* were collected (Fig. 1a, third column). *G. p. palpalis* co-existed only with a low number (3) of *Glossina* sp. in Cross River.

Of the 1802 flies collected, 391 were alive and used for dissection. Of all flies collected, the sex ratio was 48.7% males to 52.3% females in *G. p. palpalis*. However, while for *G. submorsitans* slightly more females were trapped, the opposite was observed for *G. tachinoides*, where about two third (68.1%) of all flies trapped were males (Fig. 1b).

The apparent fly densities were 23.6 F/T/D in Yankari Game Reserve, 22.5 F/T/D in Kainji Lake National Park, 13.8 F/T/D in Old Oyo National Park and 1.0 F/T/D in Cross River National Park. Apparent density of fly population in Ijah Gwari was 4.0 F/T/D.

Further trappings took place in March and December 2016 in Cameroon, Dodeo. All flies caught were *G. p. palpalis*. The sex distribution was close to equal between females (47%) and males (53%).

### Molecular analysis of COI

**Fig. 2:**
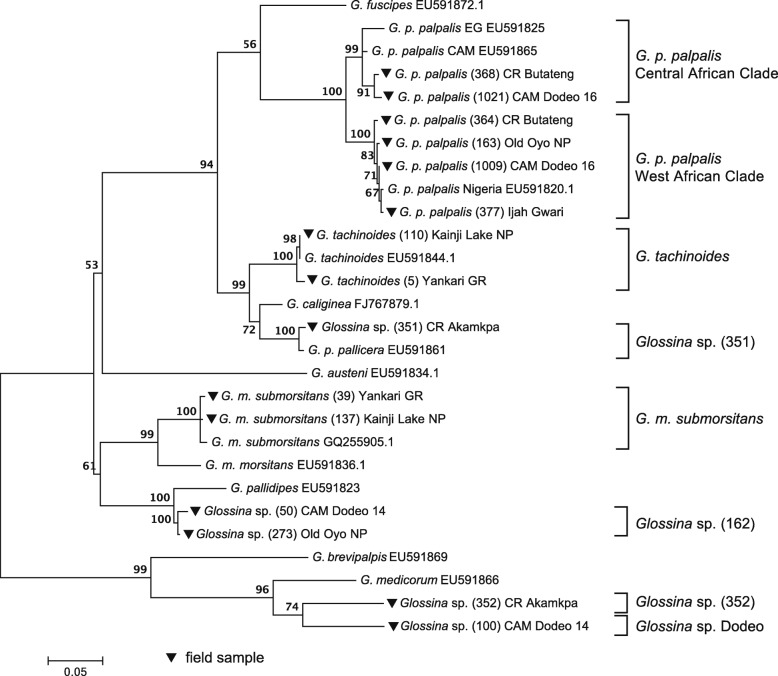
Phylogenetic analysis of *Glossina* sp. field samples (▼) and reference *Glossina* species by comparison of partial COI sequences. 592 bp stretches of representative samples for each species and sampling spot were compared to sequences obtained from NCBI as described under Methods. Tamura-Nei-93-model assuming gamma distribution and invariant sites was used to construct the tree and was evaluated with 900 bootstrap replicates. Accession numbers of reference sequences are indicated after the species name. EG Equatorial Guinea, CAM Cameroon, CR Cross River National Park

**Fig. 3:**
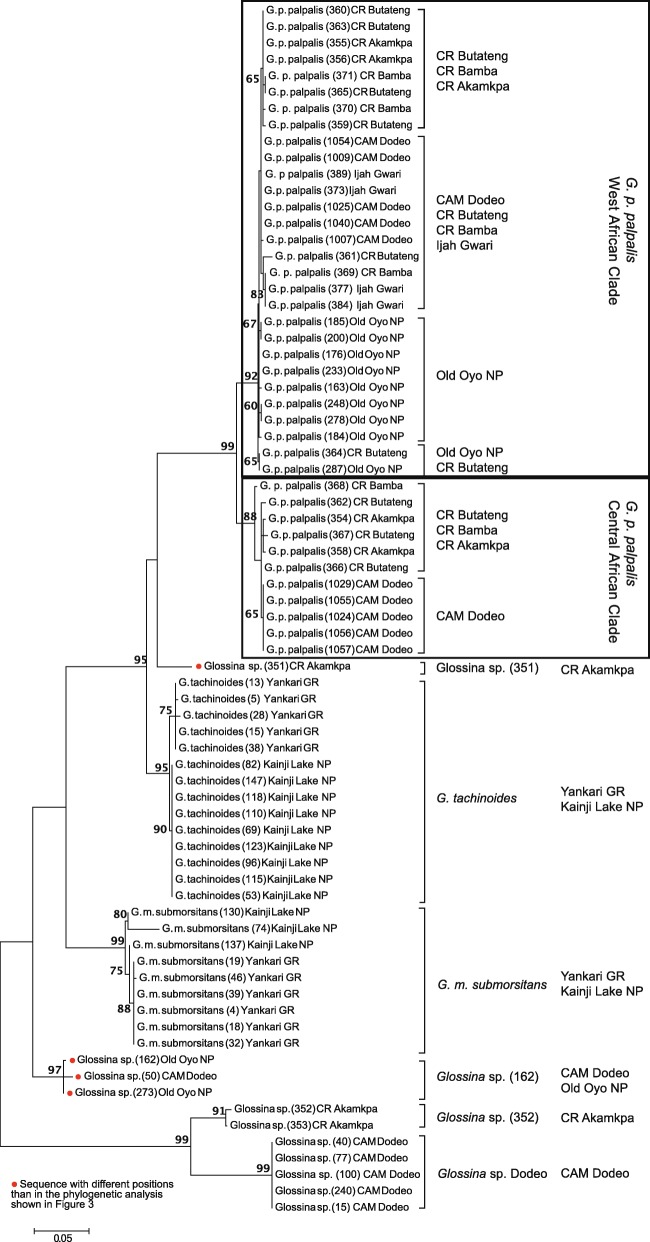
Maximum likelihood tree of partial COI sequences from *Glossina* sp. field samples. 563 bp stretches of 74 sequences were aligned and analysed as described under Methods to provide an overview of the population diversities in the different sampling sites. Tamura-3-parameter model with gamma distribution with the default setting for number of discrete gamma categories was used to construct the tree and evaluated with 800 bootstrap replicates. Sampling sites are indicated in brackets. Sequences with different positions than in the phylogenetic analysis shown in Fig. 2 are indicated. CAM Cameroon [32]

COI sequencing was done to analyse overall population structure among the different survey locations. A 900 bp fragment was amplified from 192 representative samples and a 592 bp (Fig. 2) or 563 bp (Fig. 3) stretch used for sequence comparison [8]. The unknown *Glossina* sp. 352 and Dodeo were used as outgroups to the other species.

The sequence analysis revealed an even larger species variability as recorded during fly trapping and dissections. Closely related species were observed to cluster together and separately from their distant cousins. Figure 2 shows selected samples of the respective species at each sampling site in comparison with COI sequences obtained from the database. The overall structure is as described previously [8], however, interestingly various genetically non-identified groups were recorded.

Interestingly, phylogenetic analysis of larger subgroups revealed the clustering of several subpopulations depending on the respective locations for some, but not all species (Fig. 3). The most striking observation was the division of *G. p. palpalis* populations along the Cameroon-Nigerian border. They cluster in two different branches, in one branch they grouped together with sequences from West African *palpalis* samples, while the others clustered with those from the Central African clade. Interestingly, all analysed *G. p. palpalis* from Old Oyo National Park and Ijah Gwari clustered together with the West African samples, indicating that the mixture of these clusters is locally restricted along the border. Interestingly, COI sequence divergence of these two clusters is 1% within the clusters, but 3.3–5.3% between them, independent of geographical origin.

While samples collected in Cross River National Park showed a higher divergence, samples from Dodeo seemed to be of a more homogeneous population in both clusters (West and Central African clades). The Old Oyo *G. p. palpalis* populations also clustered slightly separate from the other of the border region and Ijah Gwari.

Interestingly, *Glossina* sp. from Cross River National Park clustered in two distinctive groups, none of which is represented in the database.

The first one, *Glossina* sp. 352, clusters away from the *palpalis* and *morsitans* group, and appears to be closely related to *G. medicorum*, most likely belonging to the *fusca* group, similar to the unidentified *Glossina* sp. Dodeo (Fig. 2) collected in an earlier study in Dodeo [32].

The second one, *Glossina* sp. 351, seems to belong to the *palpalis* group (Figs. 2 and 3). However, its position within the branch is not well defined.

Furthermore, two specimens morphologically identified as *G. m. submorsitans* and caught in Old Oyo clustered together with an unidentified *Glossina* sp. (50) from Cameroon (Fig. 2, *Glossina* sp. 162). As for *Glossina* sp. 351, its position within the *Glossina* species cannot be clearly stated, in respect to the reference sequences it groups together in the *morsitans* group unexpectedly closely together with *G. pallidipes* (Fig. 2). However, this relation was not observed in the maximum likelihood analysis of the field sequences only (Fig. 3).

### ITS-1 analysis

The two *G. p. palpalis* clusters and various unidentified *Glossina* sp. were further analysed regarding changes in their ITS-1 regions [8].

The ITS-1 regions of the *G. p. palpalis* subpopulations did not show any differences and were very homogeneous. In the case of *Glossina* sp. Dodeo, the marker could be used to detect differences in the populations. In contrast to the homogeneous population indicated by the COI analysis, ITS-1 comparison grouped the sequences into two branches (data not shown). The observed differences are due to very distinct nucleotide deletions as well as several substitutions.

### Geometric wing morphometrics

**Fig. 4:**
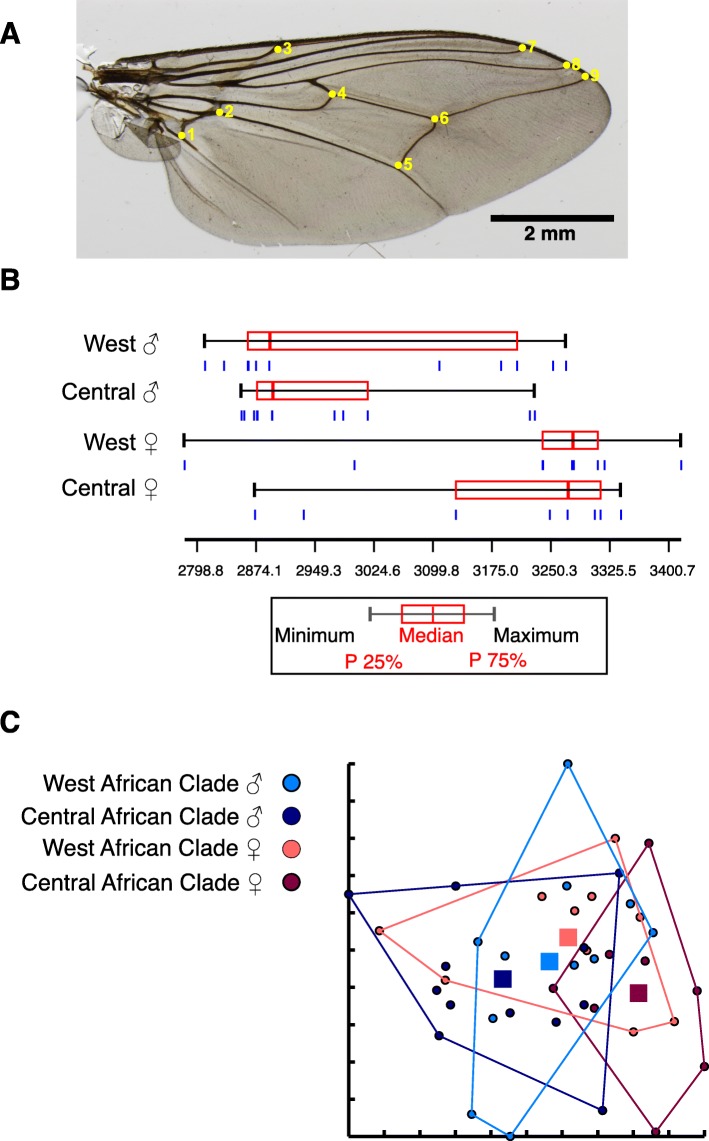
Geometric wing morphometric analysis of *G. p. palpalis* wings. (**a**) The nine landmarks selected for analysis as described previously [37] are indicated on an example wing. Centroid size (**b**) and principle component analysis of partial warps (**c**) were analysed as described under Methods. The four groups representing males and females from the Central and West African clades are indicated

Analysis of 9 landmarks was performed as previously described [37] on 41 tsetse fly wings from *G. p. palpalis* of the two genetic clusters obtained by COI analysis. The selected landmarks are shown in Fig. 4a. Due to the low number of samples collected in Nigeria’s Cross River National Park, samples collected in Dodeo, Cameroon, were used for this analysis. COI sequences from all these flies were analysed and grouped in the two observed clusters. Twenty flies belonged to the Central African cluster, while 21 samples grouped in the West African cluster.

The centroid size was used as a measure of geometric size [38] and the distance indicated a difference in the two genetic clusters in the total sample, with the Central African group showing a smaller median than the West African group. However, when comparing the principal component analysis (PCA) of the partial warps, no difference could be seen between the centroid sizes of the two populations (data not shown).

When sex distribution of the subgroups was taken into account and the samples were split accordingly, the samples showed a sex dependent shape dimorphism (Fig. 4b). The centroid distance was larger in female flies. Interestingly, PCA on the partial warps indicated that the female subgroup of the Central African cluster segregates from the others (Fig. 4c).

## Discussion

### Tsetse fly species diversity and distribution

Increased understanding in the population structure of *Glossina* species in the different vegetation zones of Nigeria will aid decisions on appropriate control strategies.

Similar fly densitites were recorded in Yankari Game Reserve and Kainji Lake National Park, while they were lower in Old Oyo National Park. During a survey in the wet season in 2012 in Yankari Game Reserve, apparent densities were 128 and 101 [39], reflecting the seasonal fluctuation compared to the apparent densities recorded in our study. The only two species identified in an earlier study [39] were *G. tachinoide*s and *G. m. submorsitans*, which corroborates our results.

While in Kainji Lake National Park only *G. m. submorsitans* and *G. tachinoides* were collected, another study found *G. p. palpalis* co-existing with *G. tachinoides* in Kainji Lake National Park [40]. This discrepancy with our results may be attributed to variations in microclimatic conditions at the sampling spots.

Interestingly, the group of the unknown *Glossina* sp. 162 matches a fly collected in Dodeo, hinting towards a ubiquitously distributed species. It will be necessary to investigate the vector capacity of these species, as they are present in various study areas and have so far not been genetically characterized. In contrast to the survey conducted in Dodeo in 2014 [32], no *Glossina* sp. Dodeo were collected during later trappings in 2016. This might be due to slightly different trap locations or the different conditions during the surveys in 2016.

### Molecular analysis of COI revealed large diversity among tsetse populations

Phylogenetic analysis using COI encoding sequences was done to observe the overall genetic species diversity and possible subpopulations within the different sampling regions. The phylogenetic tree resembles the *Glossina* species structure described previously [8], although only one mitochondrial marker was used (Fig. 2). *G. m. submorsitans* forms a group distinct from *G. tachinoides* and *G. p. palpalis*. *Glossina* sp. 352 and *Glossina* sp. Dodeo group further away from the main tree, close to the *fusca* group, and were used as outgroups.

Overall, *G. m. submorsitans* showed a slight clustering in subpopulations according to the sampling site (Fig. 3). The population in Yankari was more homogeneous and clustered away from Kainji Lake. The opposite was observed for *G. tachinoides*, where the population from Kainji Lake appears to be more homogenous than the population in Yankari, which appeared more diverse. In a study on *G. tachinoides* population structure in Burkina Faso, the homogenous population structure over a large area has been attributed to less restricted migration because of a broader temperature and humidity tolerance [41]. The observed small differences between the populations of *G. m. submorsitans* and *G. tachinoides* could therefore be due to environmental factors that may be restricting or facilitating dispersal of the respective *Glossina* species. This is indicated by the average temperature of 32.5 °C and 32% relative humidity in Yankari, which are favourable conditions for savannah species like *G. m. submorsitans*. On the contrary, in Kainji Lake a higher relative humidity of 49% at an average temperature of 31.8 °C was recorded.

In contrast to the *Glossina* species discussed above, the *G. p. palpalis* populations showed a very distinct divergence. It has been described previously that *G. p. palpalis* populations are very divergent and it has repeatedly been suggested that they represent a species complex [4, 8, 15, 23, 42]. In previous studies, genetic analysis showed a clear grouping in Central and West African clades, which has been suggested to be a process of sub-speciation [8, 15, 42]. Interestingly, also in this study we observed the same clustering, but not in geographically isolated populations, as described previously [8, 15, 23, 42], but for flies at the same location, even within the same trap. While in Central and Northwest Nigeria all *G. p. palpalis* collected group together with reference sequences of the West African Clade, all flies collected at the Nigeria-Cameroon border region segregated in two clades, the West and Central African Clade. This is especially interesting, as it has already been suggested that there might be subspecies of *G. p. palpalis* existing based on samplings in West and Central Africa [15]. In Equatorial Guinea, two geographically separated clades have been described, the island clade clustering with the West African, the main land clade with the Central African *G. p. palpalis* group [24]. The divergence of *G. p. palpalis* in Cameroon has been already observed by Dyer et al. [8], but has up to now not been investigated in detail.

Another interesting observation has been the occurrence of unidentified *Glossina* sp. In the context of vector control programs, it is very important to have a complete knowledge about all the vector species present. However, our data shows that there are still many gaps in molecular analysis, in particular for less abundant *Glossina* species. The finding of four different groups of not well-described *Glossina* species further indicates high species diversity in the forest species along the belt between the two countries Nigeria and Cameroon. The occurrence of *Glossina* sp. 162 in Old Oyo and in Dodeo indicates a wide distribution of this species in Nigeria and across the border region.

To completely resolve and correctly identify the four species clusters observed in this study, it would be necessary to collect more specimens and accurately describe them morphologically as well as to characterise them genetically.

### ITS-1 analysis

The ITS-1 region is characterized by a high variability and can thus indicate more recent changes within species and even sufficiently isolated populations. However, in the case of *G. p. palpalis* we did not identify any differences in the populations at the Nigerian-Cameroonian border. This inability of ITS-1 to distinguish *G. p. palpalis* at the population level has also been observed by others [8]. The phylogenetic analysis revealed a homogenous population. This might indicate that the segregation in two groups observed by looking at COI resembles two different maternal lineages, but not clades segregating into distinct species.

### Geometric wing morphometrics

Wing landmarks were chosen according to [37], in which subpopulations of *G. m. submorsitans* were analysed for segregation. The sexual dimorphism observed here has also been described previously for *G. p. gambiensis* [43]. Principal component analysis of the partial warps indicates a segregation of the female population belonging to the Central African group. However, due to the low sample size available for analysis, no final conclusion can be drawn and it remains to be seen whether this can be reproduced with a larger sampling size. It is interesting that the observed difference is not detectable, if data from male and female flies are pooled together.

As the divergence of *G. p. palpalis* is strongly supported by COI analysis, it is important to acknowledge this factor in breeding and vector competence, as it reflects the maternal lineage. It was shown in breeding experiments with *G. m. centralis* that susceptibility to trypanosomal infection is maternally inherited [44]. A sex dependent breeding preference has been observed and sterility in tsetse fly males has been linked to sex-chromosomal factors [45, 46]. It has been proposed that this might be due to chromosomal incompatibility [46]. Interestingly, in that same study a breeding barrier was observed between *G. p. palpalis* colonies originating from Nigeria and Zaire (Democratic Republic of Congo), while they also had different vector capacities. Similarly, in a study comparing the performance of *G. pallidipes* originating from two allopatric Kenyan populations, one from the Shimba Hills and the other from the Nguruman, it was observed that bionomic parameters did not differ between the two [47]. However, fecundity of the Shimba Hills colony females was significantly greater than that of the Nguruman colony. In addition, Nguruman culture flies were more likely to develop mature infections [48]. Furthermore, different feeding frequencies of female *G. pallidipes* from allopatric Kenya populations was demonstrated in an earlier study [49]. Differences were also recorded in activity patterns and responses to temperature [50]. In a separate study, it was shown that *G. pallidipes* cultures established from the Lambwe and from Kibwezi Forest populations differed significantly in the duration of copulation [51].

Taken together, these observations bring up the questions, if the coexisting clusters observed in this study are actually representing subspeciation or even subspecies in the classical sense in *G. p. palpalis*, and if so, can these flies interbreed or do they exhibit different breeding preferences or breeding incompatibility as observed by [46] and is there a difference in vector competence regarding trypanosomal infections between these clusters. These questions have to be investigated closely to see whether gene flow occurs between these clusters. Answering these questions is expected to generate crucial information for the application of SIT or paratransgenesis approaches.

## Conclusion

This study revealed a large diversity of *Glossina* species in Nigeria and across the border to Cameroon. The most striking observation is the coexistence of two distinctively differentiated *G. p. palpalis* groups along the Nigerian-Cameroonian border. Further characterization of these tsetse populations and dynamics is important with regard to area-wide eradication of *Glossina* species using SIT. These studies should provide information on the status of isolation of these fly populations, which would aid decision on whether control would be implemented simultaneously or continuously following the principle of the “rolling carpet” [52].

Of equal importance is the need to investigate the status of cryptic *Glossina* species or other less abundant vectors for trypanosomes in Nigeria in view of the presence of closely related sibling species, which are geographically distributed distinctly in the country.

## Abbreviations

AAT: Animal African Trypanosomosis
COI: Cytochrome C Oxidase SU1
GR: Game Reserve
HAT: Human African Trypanosomosis
ITS-1: Internal Transcribed spacer 1
NP: National Park
PCA: Principal component analysis
SIT: Sterile Insect Technique

## References

[1] Simarro PP, Diarra A, Ruiz Postigo JA, Franco JR, Jannin JG. The human African trypanosomiasis control and surveillance Programme of the World Health Organization 2000–2009: the way forward. Aksoy S, editor. PLoS Negl Trop Dis Public Libr Sci; 2011;5:e1007. 10.1371/journal.pntd.0001007 PMC3042999 21364972

[2] Franco JR, Simarro PP, Diarra A, Jannin JG (2014). Epidemiology of human African trypanosomiasis. CLEP.

[3] Swallow BM. Impacts of trypanosomiasis on African Agriculture PAAT Technical and Scientific Series, 2: Rome: FAO; 2000.

[4] Ravel S, de Meeus T, Dujardin JP, Zézé DG, Gooding RH, Dusfour I (2007). The tsetse fly Glossina palpalis palpalis is composed of several genetically differentiated small populations in the sleeping sickness focus of Bonon, Côte d’Ivoire. Infect Genet Evol.

[5] Gouteux JP (1987). A new Glossina from the Congo: *Glossina* (Austenina) *frezili* sp. nov. (Diptera: Glossinidae). Trop Med Parasitol.

[6] Pollock JN. Training manual for tsetse control personnel Vol.1 Editor: FAO Publ. No. M/P5178/E. 280p. 1982.

[7] KRAFSUR E (2009). Tsetse flies: Genetics, evolution, and role as vectors. Infection, Genetics and Evolution.

[8] Dyer NA, Lawton SP, Ravel S, Choi KS (2008). Molecular phylogenetics of tsetse flies (Diptera: *Glossinidae*) based on mitochondrial (COI, 16S, ND2) and nuclear ribosomal DNA sequences, with an emphasis on the *palpalis* group. Mol Pylogenet Evol.

[9] Leak SGA. Tsetse biology and ecology: their role in the epidemiology and control of trypanosomosis. Wallingford: CAB; 1999.

[10] Jordan AM, Service MW (1989). Man and changing patterns of the African trypanosomiases. Demography and vector-borne diseases.

[11] Abila PP, Slotman MA, Parmakelis A, Dion KB, Robinson AS, Muwanika VB (2008). High levels of genetic differentiation between Ugandan *Glossina fuscipes fuscipes* populations separated by Lake Kyoga. King CH, editor. PLoS Negl Trop Dis.

[12] Vreysen MJB, Saleh KM, Ali MY, Abdulla AM, Zhu Z-R, Juma KG, et al. *Glossina austeni* (Diptera: Glossinidae) eradicated on the island of Unguja, Zanzibar, Using the Sterile Insect Technique ec 2000;93:123–135. 10.1603/0022-0493-93.1.123 14658522

[13] Allsopp R (2001). Options for vector control against trypanosomiasis in Africa. Trends Parasitol.

[14] Proceedings of the FAO Panel of Experts (1992). Programme for the control of African animal trypanosomiasis and related development.

[15] Dyer NA, Furtado A, Cano J, Ferreira F, Odete Afonso M, Ndong-Mabale N (2009). Evidence for a discrete evolutionary lineage within Equatorial Guinea suggests that the tsetse fly *Glossina palpalis palpalis* exists as a species complex. Mol Ecol.

[16] Gooding RH, Krafsur ES (2005). Tsetse genetics: contributions to biology, systematics, and control of tsetse flies. Annu Rev Entomol.

[17] Krafsur ES (2003). Tsetse fly population genetics: an indirect approach to dispersal. Trends Parasitol.

[18] Aksoy S (2003). Control of tsetse flies and trypanosomes using molecular genetics. Vet Parasitol.

[19] Hendrichs J, Vreysen MJB, Enkerlin WR, Cayol JP (2005). Strategic Options in Using Sterile Insects for Area-Wide Integrated Pest Management. Sterile Insect Technique.

[20] Torr SJ, Maudlin I, Vale GA (2007). Less is more: restricted application of insecticide to cattle to improve the cost and efficacy of tsetse control. Med Vet Entomol.

[21] Kaba D, Ravel S, Acapovi-Yao G, Solano P, Allou K, Bosson-Vanga H (2012). Phenetic and genetic structure of tsetse fly populations (*Glossina palpalis palpalis*) in southern Ivory Coast. Parasit Vectors.

[22] Kato AB, Hyseni C, Okedi LM, Ouma JO, Aksoy S, Caccone A (2015). Mitochondrial DNA sequence divergence and diversity of Glossina fuscipes fuscipes in the Lake Victoria basin of Uganda: implications for control. Parasit Vectors.

[23] Melachio TT, Simo G, Ravel S, De MeEüs T, Causse S, Solano P (2011). Population genetics of *Glossina palpalis palpalis* from central African sleeping sickness foci. Parasit Vectors.

[24] Cordon-Obras C, Cano J, Knapp J, Nebreda P, Ndong-Mabale N, Ncogo-Ada PR (2014). *Glossina palpalis palpalis* populations from Equatorial Guinea belong to distinct allopatric clades. Parasit Vectors.

[25] Solano P, La Rocque de S, Cuisance D, Geoffroy B, de Meeus T, Cuny G (1999). Intraspecific variability in natural populations of *Glossina palpalis gambiensis* from West Africa, revealed by genetic and morphometric analyses. Med Vet Entomol.

[26] Ouma JO, Marquez JG, Krafsur ES (2007). Macrogeographic population structure of the tsetse fly, *Glossina pallidipes* (Diptera: *Glossinidae*). Bull Entomol Res.

[27] Daniel AD, Joshua RA, Kalejaiye JO, Dada AJ (1994). Prevalence of trypanosomiasis in sheep and goats in a region of northern Nigeria. Revue délevage et de médecine vétérinaire des pays tropicaux.

[28] Obaloto OB, Shamaki BU, Idehen CO, Eche TA, Balak GG, Dongkum C (2015). Survey of animal trypanosomosis and biting flies flies in parts of Alkaleri local government area, Bauchi state, Nigeria. J Biol Agr Healthc.

[29] Ngutor KS, Lawal IA, Okubanjo OO (2016). Feeding patterns and Xenomonitoring of trypanosomes among tsetse flies around the Gashaka-Gumti National Park in Nigeria. J Parasitol Res.

[30] Squarre D, Kabongo I, Munyeme M, Mumba C, Mwasinga W, Hachaambwa L (2016). Human African trypanosomiasis in the Kafue National Park, Zambia. PLoS Negl Trop Dis.

[31] Fashae O, Olusola A, Adedeji O (2017). Geospatial analysis of changes in vegetation cover over Nigeria. Bull Geog Phys Geog Ser.

[32] Ngomtcho SCH, Weber JS, Ngo Bum E, Gbem TT, Kelm S, Achukwi MD (2017). Molecular screening of tsetse flies and cattle reveal different Trypanosoma species including T grayi and T theileri in northern Cameroon. Parasit Vectors.

[33] National Parks of Nigeria. A publication of National Park Service Abuja. Nigeria: Haligraph printers Minna; 2004.

[34] Challier A, Laveissiere C (1973). Un nouveau piège pour la capture des glossines (Glossina : Diptera, Muscidae): description et eassais sur le terrain. Ent méd et Parasitol.

[35] Davies H (1977). Tsetse flies in Nigeria: a handbook for junior control staff.

[36] The CLIC Package - MoMe-CLIC. Available from: http://xyom-clic.eu/clic-collection/. Accessed on 01.01.2017, 1:59 pm.

[37] Achukwi MD, Gillingwater J, Nloga AMN, Simo G (2013). Lack of evidence for sufficiently isolated populations of *Glossina morsitans submorsitans* on the Adamawa plateau of Cameroon following geometric morphometric analysis. Adv Entomol.

[38] Rohlf FJ, Loy A, Corti M (1996). Morphometric analysis of Old World *Talpidae* (Mammalia, Insectivora) using partial-warp scores. Syst Biol.

[39] Isaac C, Ciosi M, Hamilton A, Scullion MK, Dede P, Igbinosa BI, Nmorsi, OPG, Masiga DC, Michael R, Turner CRM. Molecular identification of different trypanosome species and subspecies in tsetse flies of Northern Nigeria Parasites and Vectors. 2016;9:301. 10.1186/s13071-016-1585-3. 10.1186/s13071-016-1585-3 PMC4877947 27216812

[40] Ajibade WA, Agbede SA. Tsetse fly species diversity in Kainji Lake National Park, Nigeria. Afr J Agric Res. 3:753–8.

[41] Koné N, Bouyer J, Ravel S, Vreysen MJB, Domagni KJ, Causse S (2011). Contrasting population structures of two vectors of African Trypanosomoses in Burkina Faso: consequences for control. PLoS Negl Trop Dis.

[42] De MeEüs T, Bouyer J, Ravel S, Solano P (2015). Ecotype evolution in *Glossina palpalis* subspecies, major vectors of sleeping sickness. Caccone a, editor. PLoS Negl Trop Dis.

[43] Camara M, Caro-riaño H, Ravel S, Dujardin J-P, Hervouet J-P, De MeEüs T (2006). Genetic and morphometric evidence for population isolation of *Glossina palpalis gambiensis* (Diptera: Glossinidae) on the Loos Islands, Guinea. J Med Entomol.

[44] Moloo SK, Kabata JM, Waweru F, Gooding RH (1998). Selection of susceptible and refractory lines of *Glossina morsitans centralis* for *Trypanosoma congolense* infection and their susceptibility to different pathogenic *Trypanosoma* species. Med Vet Entomol.

[45] Gooding RH (1997). Genetic analysis of hybrid sterility in crosses of the tsetse flies *Glossina palpalis palpalis* and *Glossina palpalis gambiensis* (Diptera: Glossinidae). Can J Zool.

[46] Gooding RH, Solano P, Ravel S (2004). X-chromosome mapping experiments suggest occurrence of cryptic species in the tsetse fly *Glossina palpalis palpalis*. Can J Zool.

[47] Moloo SK (1992). A comparison of colony performance of *Glossina pallidipes originating* from two allopatric populations in Kenya. Med Vet Entomol.

[48] Moloo SK (1993). A comparison of susceptibility of two allopatric populations of *Glossina pallidipes* for stocks of *Trypanosoma congolense*. Med Vet Entomol.

[49] van Etten J (1982). Comparative studies on fat reserves, feeding and metabolic strategies of flies from two allopatric populations of *Glossina pallidipes* Austen in Kenya. Acta Trop.

[50] van Etten J (1982). Comparative studies on the diurnal activity pattern in two field and laboratory populations of *Glossina pallidipes*. Entomol Exp Appl.

[51] Jaenson TGT (1978). Mating behaviour of *Glossina pallidipes* Austen (Diptera, *Glossinidae*): genetic differences in copulation time between allopatric population. Entomol Exp Appl.

[52] Vreysen MJB, Gerardo-Abaya J, Cayol JP. Lessons from area-wide integrated pest management (AW-IPM) programmes with an SIT component: an FAO/IAEA perspective. In: Vreysen MJB, Robinson AS, Hendrichs J, editors. Area-Wide Control of Insect Pests: From Research to Field Implementation. Dordrecht: Springer; 2007:723–44.

